# Self-administration of gender-affirming hormones: a systematic review of effectiveness, cost, and values and preferences of end-users and health workers

**DOI:** 10.1080/26410397.2022.2045066

**Published:** 2022-03-21

**Authors:** Caitlin E. Kennedy, Ping Teresa Yeh, Jack Byrne, L. Leigh Ann van der Merwe, Laura Ferguson, Tonia Poteat, Manjulaa Narasimhan

**Affiliations:** a Associate Professor, Department of International Health, Johns Hopkins Bloomberg School of Public Health, Baltimore, MD, USA; b Research Associate, Department of International Health, Johns Hopkins Bloomberg School of Public Health, Baltimore, MD, USA; c Co-investigator, Counting Ourselves, Trans Health Research Lab, the Aotearoa New Zealand Trans and Non-binary Health Survey, University of Waikato, Hamilton, New Zealand; d Founder/Director & Research Lead, Social, Health and Empowerment Feminist Collective of Transgender Women of Africa, East London, South Africa; e Assistant Professor, Institute on Inequalities in Global Health, Keck School of Medicine, University of Southern California, Los Angeles, CA, USA; f Associate Professor, Department of Social Medicine, University of North Carolina School of Medicine, Chapel Hill, NC, USA; g Scientist, Department of Sexual and Reproductive Health and Research, World Health Organization, includes the UNDP/UNFPA/UNICEF/WHO/World Bank Special Programme of Research, Development and Research Training in Human Reproduction – HRP, Geneva, Switzerland

**Keywords:** gender-affirming hormone therapy, self-care, transgender health, self-administration, values and preferences, systematic review

## Abstract

Self-administration of quality gender-affirming hormones is one approach to expanding access to hormone therapy for individuals seeking secondary sex characteristics more aligned with their gender identity or expression and can be empowering when provided within safe, supportive health systems. To inform World Health Organization guidelines on self-care interventions, we systematically reviewed the evidence for self-administration compared to health worker-administration of gender-affirming hormones. We conducted a comprehensive search for peer-reviewed articles and conference abstracts that addressed effectiveness, values and preferences, and cost considerations. Data were extracted in duplicate using standardised forms. Of 3792 unique references, five values and preferences articles were included; no studies met the criteria for the effectiveness or cost reviews. All values and preferences studies focused on self-administration of unprescribed hormones, not prescribed hormones within a supportive health system. Four studies from the U.S. (*N* = 2), Brazil (*N* = 1), and the U.K. (*N* = 1) found that individuals seeking gender-affirming hormone therapy may self-manage due to challenges finding knowledgeable and non-stigmatising health workers, lack of access to appropriate services, exclusion, and discomfort with health workers, cost, and desire for a faster transition. One study from Thailand found health worker perspectives were shaped by restrictive legislation, few transgender-specific services or guidelines, inappropriate communication with health workers, and medical knowledge gaps. There is limited literature on self-administration of gender-affirming hormone therapy. Principles of gender equality and human rights in the delivery of quality gender-affirming hormones are critical to expand access to this important intervention and reduce discrimination based on gender identity.

## Résumé

L’auto-administration d’hormones de qualité pour l’affirmation du genre est une méthode permettant d’élargir l’accès au traitement hormonal des individus souhaitant des caractéristiques sexuelles secondaires plus alignées sur leur identité ou leur expression de genre; elle peut les autonomiser quand ce traitement est prodigué dans le cadre de systèmes de santé sûrs et bienveillants. Pour contribuer aux lignes directrices de l’Organisation mondiale de la santé sur les interventions d’auto-prise en charge, nous avons examiné de manière systématique les données sur l’auto-administration en les comparant aux hormones d’affirmation du genre administrées par des personnels de santé. Nous avons mené une recherche complète d’articles publiés dans des revues à comité de lecture et de résumés de conférences qui abordaient l’efficacité, les valeurs et les préférences, ainsi que des considérations de coût. Les données ont été extraites en double exemplaire à l’aide de formulaires normalisés. Des 3792 références uniques, cinq articles sur les valeurs et les préférences ont été inclus; aucune étude ne réunissait les critères pour l’examen de l’efficacité ou du coût. Toutes les études sur les valeurs et les préférences se centraient sur l’auto-administration d’hormones sans ordonnance, des hormones qui n’avaient pas été prescrites dans le cadre d’un système de soins de santé bienveillant. Quatre études venant des États-Unis (N = 2), du Brésil (*N* = 1) et du Royaume-Uni (*N* = 1) ont révélé qu’il est possible que des individus recherchant un traitement hormonal d’affirmation du genre s’auto-prennent en charge en raison des difficultés à trouver des agents de santé compétents qui ne les stigmatisent pas, du manque d’accès aux services appropriés, de l’exclusion et du malaise avec les personnels de santé, du coût et du souhait d’une transition plus rapide. Une étude portant sur la Thaïlande a montré que les perspectives des agents de santé étaient façonnées par la législation restrictive, les rares services ou directives propres aux transgenres, la communication inadaptée entre prestataire et patient, et les lacunes dans les connaissances médicales. On dispose de publications limitées sur l’auto-administration de traitement hormonal d’affirmation du genre. Les principes de l’égalité des genres et des droits de l’homme dans l’administration d’hormones de qualité pour l’affirmation du genre sont essentiels afin d’élargir l’accès à cette importante intervention et de réduire la discrimination fondée sur l’identité de genre.

## Resumen

La autoadministración de hormonas de calidad que reafirman el género es un enfoque para ampliar el acceso a la terapia hormonal para personas que buscan características sexuales secundarias más alineadas con su identidad o expresión de género, y puede empoderarlas cuando se realiza dentro de sistemas de salud seguros que brindan apoyo. Con el fin de informar la guía de la Organización Mundial de la Salud sobre intervenciones de autocuidado, revisamos sistemáticamente la evidencia de autoadministración de hormonas que reafirman el género, comparada con la administración por trabajadores de salud. Realizamos una búsqueda integral de artículos revisados por pares y resúmenes de conferencias que tratan consideraciones de eficacia, valores y preferencias, y costo. Se extrajeron datos en duplicado utilizando formularios normalizados. De 3792 referencias únicas, se incluyeron cinco artículos sobre valores y preferencias; ningún estudio reunió los criterios para las revisiones de eficacia o costo. Todos los estudios de valores y preferencias se enfocaron en la autoadministración de hormonas no recetadas, y no en hormonas recetadas dentro de un sistema de salud que brinda apoyo. Cuatro estudios realizados en Estados Unidos (*N* = 2), Brasil (*N* = 1) y el Reino Unido (*N* = 1) encontraron que las personas que buscan terapia hormonal que reafirma el género podrían autogestionarla debido a los retos de encontrar trabajadores de salud bien informados y no estigmatizantes, la falta de acceso a servicios adecuados, la exclusión y la incomodidad con trabajadores de salud, costo y deseo de una transición más rápida. Un estudio de Tailandia encontró que en las perspectivas de los trabajadores de salud influían legislación restrictiva, pocos servicios o lineamientos específicos para personas transgénero, comunicación inadecuada entre trabajadores de saludy pacientes, y brechas de conocimiento médico. Existe literatura limitada sobre la autoadministración de terapia hormonal que reafirma el género. Los principios de igualdad de género y derechos humanos en la entrega de hormas de calidad que reafirman el género son fundamentales para ampliar el acceso a esta importante intervención y reducir la discriminación basada en la identidad de género.

## Background

Recent years have seen substantially increased commitment to understanding and improving the health and well-being of the estimated 25 million transgender people and other gender minorities globally.^1^ Holistic care for transgender and gender-diverse individuals is critical, yet too often unavailable globally. To affirm their gender identity, individuals may seek out a range of interventions and behavioural adaptations, including hormone therapy, surgery, facial hair removal, speech and communication interventions, genital tucking or packing, and chest binding.^2^ Health systems must be designed to support individuals to seek those interventions which they desire to affirm their gender identity. Support for gender-affirming interventions should be part of an overall supportive structure that ensures that no additional harm, marginalisation, stigma, or discrimination is caused to transgender and gender-diverse individuals who are too often ill-served by health care systems.

Gender-affirming hormone therapy is one gender-affirming intervention which enables the acquisition of secondary sex characteristics more aligned with an individual's gender identity or expression.^2^ In gender-affirming hormone therapy, a range of hormones may be administered in several ways, such as through injection, ingestion (oral), insertion of implants, and topical application (creams, gels or patches). Gender-affirming hormone therapy has been defined as medically necessary by the World Professional Association for Transgender Health,^3^ although it is not acknowledged as medically necessary in many settings. A recent systematic review of the literature found that hormone therapy was associated with increased quality of life, decreased depression, and decreased anxiety, although confidence in the causal nature of these associations was limited as existing studies have relatively high potential for bias in study design, have small sample sizes, and may be unable to separate the effects of multiple interventions.^4^

Ideally, gender-affirming hormone therapy would take place in the context of a supportive health system. However, many transgender and gender-diverse individuals do not have access to such a supportive system. Instead, they may seek to access such therapy through friends, peers, and the internet, without consulting a health worker.^5–8^ Several quantitative studies of transgender and gender-diverse individuals globally have documented reported rates of unprescribed hormone use ranging from 11% in Ontario, Canada,^9^ to 31% in London, United Kingdom,^10^ to 49.1% in San Francisco, United States,^11^ to 78.7% in Rio de Janeiro, Brazil.^12^ While some routes of administration may be used fairly easily by an individual without health system support, others may pose risks; at least one study has suggested that self-injection compared to no injection is associated with increased prevalence of HIV, perhaps due to sharing needles.^13^ Nevertheless, even (or perhaps particularly) in the context of an unsupportive health system, there may be better ways to support individuals to safely and effectively self-administer gender-affirming hormones.

The World Health Organization (WHO) defines self-care as “the ability of individuals, families and communities to promote health, prevent disease, maintain health, and cope with illness and disability with or without the support of a health worker”.^14^ Self-administration of quality gender-affirming hormones is one approach to expanding access to hormone therapy for those who desire it, and it can be empowering when provided within safe, supportive health systems. There are several possible ways in which individuals could self-administer hormones, which vary by route of administration. For injectable hormones, self-administration may refer to self-injection rather than injection administered by a health worker. For oral or topical forms of hormones, self-administration may refer to self-prescription, as these formulations are already commonly taken or applied by the user. Appropriate approaches to self-administration will vary depending on the type of hormone and form of administration. For example, some injectable formulations may be readily self-administered (i.e. (bi)weekly testosterone, oestradiol valerate), while others may not be (i.e. long-acting testosterone undecanoate, testosterone pellets).

Expanding access to self-administration of gender-affirming hormones may help support a rational distribution of tasks across clients and health workers, thus potentially expanding the ability of the health system to offer access to the benefits of such therapy. For clients, self-administration may also be more efficient and convenient, offering the possibility of fewer health facility visits; more private; and more empowering, facilitating greater client control over their own bodies and health. It also may enable safer use of such therapies in settings where transgender and gender-diverse individuals face discrimination and violence.

The goal of this systematic review was to assess the evidence for self-administration compared to health worker-administration of gender-affirming hormones, as an additional option, ideally within the structure of a safe and supportive health system. Specifically, we defined self-administration as related to either self-prescription (in the case of oral or topical hormones) or self-injection (in the case of injectable hormones).

We conducted this review in the context of expanding the evidence base of the 2019 WHO guideline on self-care interventions^14^ to include interventions that support gender-affirming care. As part of this guideline, community workshops with transgender people around self-care highlighted the issue of self-administration of gender-affirming hormones.^15^ These consultations noted both benefits and barriers to self-care. Benefits included the fact that educated individuals can access health information online, allowing them to manage interventions on their own and guide their peers, and the fact that there is no stigma or fear of disclosure in accessing self-care products; barriers included the lack of a support system or remedial measures in case of failure or complications. While currently there are no WHO recommendations on gender-affirming hormone therapy, consensual gender-affirming treatments and therapies (organised by oral, topical, and parenteral hormone therapy) are listed in the WHO Universal Health Care compendium for early adolescence through late adulthood.^16^

We also conducted this review in the context of the need to maintain essential health services during the COVID-19 pandemic, which has seen overstretched health systems, interruptions in the supply of gender-affirming products and disrupted services due to country-wide lockdowns globally.^17^ Qualitative interviews with lesbian, gay, bisexual, transgender, intersex, and queer individuals during the COVID-19 pandemic confirmed that, among other challenges, some of these individuals are experiencing increased disruptions in health care access and reluctance to seek care due to the pandemic.^18,19^ In particular, during health emergencies, transgender and gender-diverse individuals may be cut off from hormones and other gender-affirming care.^20^

## Methods

This review addressed the following question: Should self-administration of gender-affirming hormones be made available in addition to health worker-administration? We defined self-administration according to the WHO self-care guideline definition as “the process of people administering pharmacological substances or biomedical interventions to themselves”.^14^ For self-administration of gender-affirming hormones, we considered either self-prescription of oral or topical hormones or self-injection of injectable hormones (either self-prescribed or health worker-prescribed hormones). We recognise that this definition includes a wide range of practices that are not comparable on many aspects. However, we decided a broad approach would capture a breadth of information for the purposes of the review.

We reviewed the extant literature in three areas relevant to the review question: effectiveness of the intervention, values and preferences of end users and health workers, and cost information. The review followed PRISMA guidelines^21^ and the protocol was published on PROSPERO (registration ID: CRD4202 1231648).

### Effectiveness review

Following the WHO Handbook for Guideline Development,^22^ the effectiveness review was designed according to the PICO format as follows:

*Population:* Individuals seeking to use gender-affirming hormones

*Intervention:* Self-administration of gender-affirming hormones (either self-prescription of oral or topical hormones or self-injection of injectable hormones).

*Comparison:* Health worker-administration of gender-affirming hormones

*Outcomes:*

(1) Correct use of hormones/hormone levels (dose)
(2) Side effects and adverse events (e.g. pituitary adenoma (prolactinoma), galactorrhea, venous thromboembolism, low libido, autoimmunity, migraine, cancer, cardiovascular health, HIV infection, Hepatitis C infection), including knowledge of potential interactions with other medications and/or experience of such interactions
(3) Trust and engagement with the health system for issues other than hormone use, or for following up with side effects, etc.
(4) Self-efficacy, empowerment, and self-determination (gender affirmation/ transgender identity expression and validation), autonomy (informed consent and decision-making around bodily autonomy), including around sexual health and sexuality (confidence, communication with partners, self-esteem)
(5) Mental health and well-being (life satisfaction, self-rated well-being, happiness, resiliency, coping, anxiety, stress, self-harm), eating disorders, substance or alcohol use
(6) Social harms (stigma, discrimination, coercion, violence [including intimate-partner violence, violence from family members or community members, etc.]), and whether these harms were corrected/had redress available
(7) Satisfaction with and appropriateness of clinical services (e.g. services that do not consider being transgender as a pathology or mental health issue, but as one form of gender diversity)
(8) Peer/community support

To be included in the effectiveness review, a study had to meet the following criteria:

1. Study design that compared self-administration of gender-affirming hormones to health worker-administration of the same gender-affirming hormones; this included both randomised trials and observational studies that compare individuals who received the intervention to those who received health worker-administration of gender-affirming hormones
2. Measured one or more of the outcomes listed above
3. Published in a peer-reviewed journal or as a conference abstract

When data were available, we planned to stratify all analyses by the following categories:

- Specific hormone regimens (e.g. testosterone, oestrogen/oestradiol, anti-androgens, progesterone) and/or route of administration (e.g. injectable, oral, topical)
- Self-administration with support of/linkage to the health system vs. self-administration without connection to the health system, including quality of hormones and access to safe injecting equipment
- Age (e.g. adolescents vs. adults)
- Point of access (e.g. stores, pharmacies, online, telehealth, friends/trans community members, etc.)
- Populations (e.g. individuals with specific medical conditions or on specific medications)
- Vulnerabilities (e.g. poverty, disability)
- High-income versus low- or middle-income countries
- Literacy/educational level

No restrictions were placed based on the location of the intervention. No language restrictions were used on the search. Articles in English, French, Spanish, and Chinese were coded directly; articles in other languages were translated into English before coding.

The following electronic databases were searched through the search date of November 4, 2020: PubMed, CINAHL, LILACS and EMBASE. No restrictions were placed on the date of publication. We searched for ongoing RCTs through clinicaltrials.gov, the WHO International Clinical Trials Registry Platform, the Pan-African Clinical Trials Registry, and the Australian New Zealand Clinical Trials Registry. We searched for conference abstracts at the following conferences: World Professional Association for Transgender Health (WPATH), USPATH (United States), CPATH (Canada), EPATH (Europe), AsiaPATH (Asia), AusPATH (Australia), PATHA (Aotearoa/New Zealand), Conference on Retroviruses and Opportunistic Infections (CROI), International AIDS Society (IAS), GLMA Health Professional Advancing LGBTQ Equality, National LGBT Health Conference, Philadelphia Trans Health Conference, and Advancing Excellence in Trans Health Conference. Secondary reference searching was conducted on all studies included in the review. Finally, selected experts in the field were contacted to identify additional articles not identified through other search methods.

We developed a comprehensive search strategy adapted to each database (Appendix 1) which we used to identify studies for the main effectiveness systematic review (PICO question) and for the values and preferences and costs reviews (described below). Titles, abstracts, citation information, and descriptor terms of citations identified through the search strategy were screened by a member of the senior study staff. Full-text articles were obtained of all selected abstracts and two independent reviewers assessed all full-text articles for eligibility to determine final study selection. Differences were resolved through consensus.

We planned to have two reviewers extract data independently using standardised data extraction forms, with referral to a senior study team member from WHO when necessary. We planned to gather the following information from each included PICO study:

- Study identification: Author(s); type of citation; year of publication
- Study description: Study objectives; location; population characteristics; type of hormones; study design; sample size; follow-up periods and loss to follow-up
- Outcomes: Analytic approach; outcome measures; comparison groups; effect sizes; confidence intervals; significance levels; conclusions; limitations

For randomised trials, we planned to assess risk of bias using the Cochrane Collaboration’s tool for assessing risk of bias.^23^ For non-randomised trials but comparative studies, we planned to assess study rigour using the Evidence Project risk of bias tool for intervention evaluations.^24^

### Values and preferences review

The same search terms were used to search and screen for studies to be included in the values and preferences review. Studies were included in this review if they presented primary data examining preferences of gender-affirming hormone users or individuals who might be candidates for gender-affirming hormone use. We focused on studies examining the values and preferences of end users but also included studies examining the values and preferences of health workers and other stakeholders.

For the values and preferences review, we included studies that focused on reasons why individuals choose to use gender-affirming hormones outside of medical supervision, and health worker perspectives on self-management of gender-affirming hormones. We excluded studies that focused on the fact that self-administration of hormones exists or reasons why individuals choose to use hormones in general. We also considered issues related to age restrictions, informed decision-making, stigma and discrimination, coercion, and seeking redress in this section. These studies could be qualitative or quantitative in nature but had to present primary data. As this was a secondary component of the review, we did not plan for a formal quality assessment of included studies. Values and preferences literature was summarised qualitatively and organised by study design and methodology, location, and population.

### Cost review

The same search strategy was used to identify studies to be included in the cost review. Studies would have been included in this review if they presented primary data comparing cost or cost-effectiveness of the intervention and comparison listed in the PICO above, or if they presented cost-effectiveness of the intervention related to the PICO outcomes listed above. As with the values and preferences review, we did not plan for a formal quality assessment of studies in the cost review. We planned to summarise cost literature qualitatively, classifying it into four categories (health sector costs, other sector costs, patient/family costs, and productivity impacts) and within each category organising by study design/methodology, location, and population.

## Results

**Figure 1.**
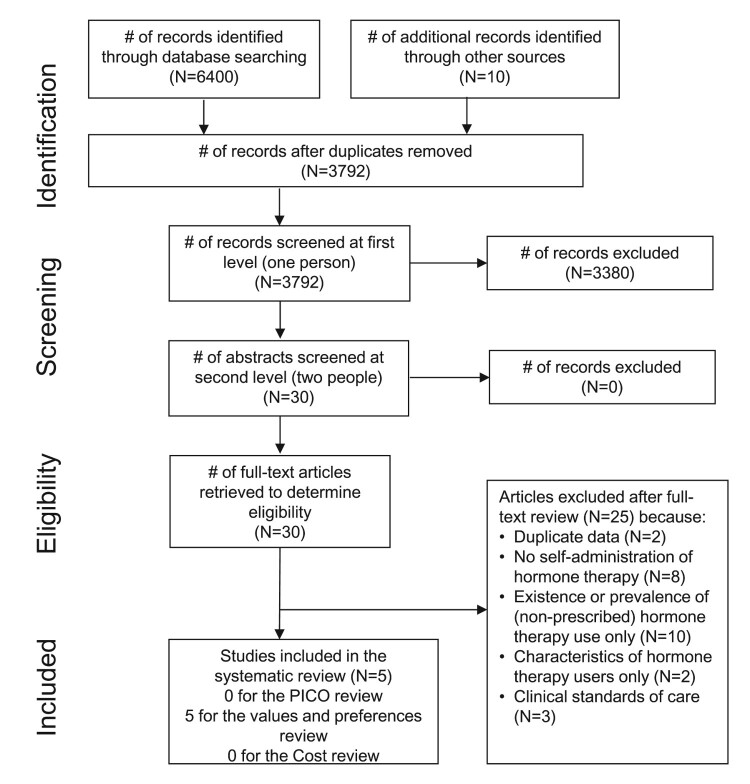
PRISMA flow chart showing disposition of citations through the search and screening process

Our search yielded 3792 unique references, of which 30 were retained for full-text review (Figure 1). Of these, 25 were excluded for not meeting our inclusion criteria (Appendix 2). Ultimately, we identified no studies that met the inclusion criteria for the effectiveness review. Therefore, quality assessment was not conducted. We also identified no studies that met the inclusion criteria for the cost review.

We did identify five studies meeting the inclusion criteria for the values and preferences review (Table 1). All were peer-reviewed articles. Two were conducted in the United States, one in Brazil, one in the United Kingdom, and one in Vietnam. All focused on self-administration of unprescribed hormones; none examined preferences related to self-administration of prescribed hormones within a supportive health system.

**Table 1.** Description of articles included in the values and preferences review^a^

Study	Location and setting	Sample size, population, age range, hormone types and modes of administration	Study design and analysis	Key findings related to hormone self-administration
de Haan et al.^11^	San Francisco, California, USA Respondent-driven sampling starting with diverse local trans individuals	314 ethnically diverse trans women aged 18+ (majority were over age 40) 68.7% were currently on hormone replacement therapy (HRT), oestrogen (62.2%), anti-androgens (38.7%), and Perlutal, an injectable contraceptive usually purchased via the internet or personal networks (12.4%); 49.1% reported taking hormones for HRT not prescribed by a clinician	Quantitative (cross-sectional survey) Descriptive and multivariable analysis	Most common reasons for taking nonprescribed hormones were not being able to see a health worker (35.5%) and wanting a quicker gender transition (12%)
Glick et al.^25^	New Orleans, Louisiana, USA Sampling through flyers and snowball recruitment	18 transgender and gender-diverse individuals aged 23–64 and 5 health workers (age not reported) Hormones (not further described), sometimes ordered online (through international pharmacies or pharmacies in other states) without a prescription, shared with friends, or stealing	Qualitative (semi-structured interviews) Thematic analysis	Reasons for taking nonprescribed hormones included challenges finding a health worker who was both trans-friendly and trans-knowledgeable, cost, and avoiding surveillance and having to interaction with the biomedical care delivery system or establish new relationships with health workers
de Andrade et al.^26^	Recife, Brazil Snowball sampling through local transgender and gender-diverse groups	10 transgender women ages 22–40 Sex hormones [not further described] not under the supervision of the study team	Qualitative (semi-structured interviews) Software-driven textual analysis	Exclusion, lack of relevant services, and lack of appropriately prepared health workers in the primary health care sector led individuals to seek gender-affirming hormones elsewhere
Metastasio et al.^27^	Suffolk (a rural county North East of London) and London (Camden and Islington Boroughs), England (United Kingdom) General adult psychiatry settings (inpatient and outpatient)	7 transgender and gender-diverse individuals ages 19–36 5 cases of oestrogens and 2 cases of androgens. One oestrogen case described oestrogen cream; the others did not describe mode of administration. Two oestrogen cases also described using finasteride (one specified tablets, the other did not specify mode of administration), and two described also using finasteride and spironolactone. Androgen cases were not further described	Clinical case report	Lengthy timelines of care in the formal sector – including long waiting lists and various steps of assessment and treatment – prompted some individuals to seek faster results by reading protocols and purchasing hormones online. Two individuals sought care online first, then asked their doctor for follow-up prescriptions. Stigma and marginalisation were also noted as potential reasons for self-administration
Do and Nguyen^28^	Hanoi and Ho Chi Minh City, Vietnam Snowball sampling of health workers at medical institutions	12 health workers (age not reported) Cross-sex hormones [not further described]	Qualitative (semi-structured interviews) Thematic analysis with themes organized by socio-ecological level	Factors that influenced the provision of medical services included: – restrictive legislation (policy level) – shortage of transgender-specific services, and lack of training and guidelines (organisational level) – ambiguous perceptions, inappropriate health worker communication, and medical knowledge gaps (individual level)

^a^ As described in the original source.

Four studies focused on the values and preferences of end users. In the United States, de Haan et al.^11^ conducted a quantitative survey among 314 ethnically diverse trans women in San Francisco. The most common reasons for taking non-prescribed hormones were not being able to see a health worker (35.5%) and wanting a quicker gender transition (12%). Also in the United States, but in New Orleans, Glick et al.^25^ conducted qualitative interviews with 18 transgender and gender-diverse individuals. They found that reasons for taking non-prescribed hormones included challenges finding a health worker who was both trans-friendly and trans-knowledgeable, cost, and avoiding surveillance and having to interact with the biomedical care delivery system or establish new relationships with health workers. In Brazil, de Andrade et al.^26^ conducted a qualitative study with 10 transgender women using hormones in Recife. They found that exclusion, lack of relevant services, and lack of appropriately prepared health workers in the primary health care sector led individuals to seek gender-affirming hormones elsewhere. Finally, in the United Kingdom, Metastasio et al.^27^ presented a case report of seven transgender and gender-diverse individuals’ reports of self-prescribing and self-administering hormones. They found that lengthy timelines of care in the formal sector – including long waiting lists and various steps of assessment and treatment – prompted some individuals to seek faster results by reading protocols and purchasing hormones online. Of the seven cases, two sought care online first, then asked their doctor for follow-up prescriptions. Stigma and marginalisation were also noted as potential reasons for self-administration.

The fifth values and preferences study, by Do et al.,^28^ was conducted among health workers in Vietnam. Using the socio-ecological framework, they found that factors that influenced the provision of medical services included restrictive legislation (policy level), a shortage of transgender-specific services, and lack of training and guidelines (organisational level), ambiguous perceptions, inappropriate health worker communication (such as misgendering and using language that assigns a higher social status to the health worker), and medical knowledge gaps (individual level). Some health workers felt there were harms resulting from hormone self-medication, but they did not explain or elaborate on what these harms might be. One said he could not possess the same level of knowledge as transgender patients: *“To be honest, they know better than we doctors do about hormones. Because they take them. While we do not have much time to do research on this topic”.*^28^

## Discussion

Our review identified no studies on effectiveness of self-administration of gender-affirming hormones. The lack of comparative effectiveness data means that clinical guidance must rely on expert opinion and anecdotal experience to determine what aspects of care can be delegated to the client and which should be retained by the health system. We also identified no studies on cost or cost-effectiveness. The lack of cost data, including the relative costs to the health system and the users for self-administration and health worker-administration, also hinders guidance development. However, the costs of gender-affirming hormone therapy are likely to vary widely depending on the type of therapy, mode of administration, length of use, source of hormones and other products, tests administered, and insurance coverage in a given health system.

While we did identify five studies reporting on values and preferences around self-administration of gender-affirming hormones, all of these discussed self-administration of unprescribed hormones; none examined preferences related to self-administration of prescribed hormones within a supportive health system. These studies suggested that self-management is related to challenges finding knowledgeable and non-stigmatising health workers, lack of access to appropriate services, exclusion, discomfort managing relationships with health workers, cost and desire for a faster transition. Health worker perspectives on self-administration are also affected by multiple factors including restrictive legislation, lack of transgender-specific services and guidelines, inappropriate provider–patient communication, and medical knowledge gaps. Transgender and gender-diverse individuals face multiple, intersecting forms of inequalities, violence, stigma, and discrimination, so gender and human rights considerations must be ensured in the development of supportive approaches to health provision.^29^

To understand the reasons behind this dearth of literature, we reflected on the relatively recent increase in appreciation for and interest in transgender health: a PubMed search for “transgender” indicates a 10-fold increase in publications over the last decade. Anecdotally, we find that clinician concerns around self-administration tend to be about either the types of hormones being used (e.g. use of oral contraceptives as hormones for gender affirmation in the Pacific and Africa; no longer approved hormones being available at drug stores in Asia; or transgender women in Asia using progesterone, contrary to recommendations, because of reported effects on the nipple areola and libido), the amount of hormones being used, or injecting techniques (e.g. sharing of needles or concerns about testosterone self-administration due to potential breathing problems or allergic reactions).^30^ Conversely, for transgender and gender-diverse individuals, self-administration tends to be framed around access to hormones when they are not available through local health systems (e.g. ordering hormones online or from other countries), access to a wider range of hormones (particularly progesterone), independence and/or informed consent, and cost (not having to pay to see a health worker to access the hormones and/or have them administered). These areas of focus for different stakeholders may lead to limited comparative research into what can be safely and effectively self-administered versus administered by a health worker. We also note that in some cases, research has examined interventions that are self-administered that differ from those that are health worker-administered. For example, research has explored efficacy and satisfaction with self-administered subcutaneous testosterone injection compared with health worker-administered intramuscular injection, finding that self-administration of subcutaneous testosterone is an effective, safe, and accepted alternative.^31^

Further, across the world, transgender and gender-diverse individuals live within social, legal, economic, and political systems that place them at high risk of discrimination, exclusion, poverty, and violence.^32^ As Connell^33^ has argued, “familiar models of professional health care” – such as clinical guidelines – “are not adequate to these issues across much of the world; social action and organising are required”.^33^ This calls for a harm reduction approach. While the idea of supportive and rights-affirming health systems that provide gender-affirming care following evidence-informed recommendations should be our goal, this is far from reality in much of the world. A harm reduction approach, which recognises that self-care may be the only option available to many individuals, may guide supportive public health approaches in these settings. The 2018 revision to the International Classification of Diseases (ICD-11), which removed “gender dysphoria” from the mental and behavioural disorders chapter and introduced “gender incongruence” to a new chapter on conditions related to sexual health, provides an opportunity for advocacy to improve laws, systems and services for transgender and gender-diverse individuals.

We also recognise that the concept of self-administration versus health worker-administration is complex and encompasses a range of different situations. In particular, “administration” is more easily defined for injectables or implants – where the health worker potentially knows what is being injected and the dose. In contrast, self-administration could raise risks in terms of the hormone used and/or how the injection was done. However, for oral or topical forms of hormones, health workers may prescribe the hormones, but are unlikely to routinely watch someone take their hormone pills or apply their gel or patch. There may also be important differences between self-initiation and self-administration of gender-affirming hormones initiated within health care settings. We kept our review broad to encompass this range of potential situations and found little. However, we recognise that guidance around self- versus health worker-administration would need to provide greater details around the specific modes of administration and initiation. Further research could delineate these different areas and document how self-administration is conceptualised by both end users and health workers across a range of hormones, doses, and experiences.

Given our findings from this review, there is limited data to answer our primary research question: Should self-administration of gender-affirming hormones be made available in addition to health worker-administration? As noted above, this question itself was broad and encompassed a range of different hormones and forms of administration which might warrant different recommendations. Based on this review’s findings, the WHO guideline review group agreed upon the following key considerations for use of self-administration of gender-affirming hormones for transgender and gender-diverse individuals:

- *“The principles of gender equality and human rights in the delivery of quality gender-affirming hormones are critical to expanding access to this important intervention and reducing discrimination based on gender identity.*
- *Transgender and gender-diverse people live within social, legal, economic and political systems that place them at high risk of discrimination, exclusion, poverty and violence.*
- *Research is urgently needed to support evidence-driven guidance.”*^34^

Strengths of this review include our broad search strategy to identify literature on a range of issues related to self-administration of gender-affirming hormones: effectiveness, values and preferences, and cost. However, our conclusions are limited by the dearth of studies that met our inclusion criteria. As we limited our review to peer-reviewed articles and conference abstracts, we may have missed information available only in the grey literature. With increased support for transgender health globally, we hope that future studies may be published to expand this evidence base to provide critical information to inform clinical care recommendations and achieve health equity for transgender and gender-diverse individuals.

## Conclusions

There is limited literature on self-administration of gender-affirming hormone therapy as an additional option to health worker-administration. Principles of gender equality and human rights in the delivery of quality gender-affirming hormones are critical to expanding access to this important intervention and reducing gender discrimination based on gender identity.

## Data Availability

All data come from published articles. Extracted data are available on request to the corresponding author.

## Appendices

### Appendix 1. PRISMA 2009 checklist.

**Table:**

**Section/topic**	#	**Checklist item**	**Reported on page #**
**TITLE**			
Title	1	Identify the report as a systematic review, meta-analysis, or both.	1
**ABSTRACT**			
Structured summary	2	Provide a structured summary including, as applicable: background; objectives; data sources; study eligibility criteria, participants, and interventions; study appraisal and synthesis methods; results; limitations; conclusions and implications of key findings; systematic review registration number.	2
**INTRODUCTION**			
Rationale	3	Describe the rationale for the review in the context of what is already known.	3–4
Objectives	4	Provide an explicit statement of questions being addressed with reference to participants, interventions, comparisons, outcomes, and study design (PICOS).	4–5
**METHODS**			
Protocol and registration	5	Indicate if a review protocol exists, if and where it can be accessed (e.g., Web address), and, if available, provide registration information including registration number.	5
Eligibility criteria	6	Specify study characteristics (e.g., PICOS, length of follow-up) and report characteristics (e.g., years considered, language, publication status) used as criteria for eligibility, giving rationale.	5
Information sources	7	Describe all information sources (e.g., databases with dates of coverage, contact with study authors to identify additional studies) in the search and date last searched.	6
Search	8	Present full electronic search strategy for at least one database, including any limits used, such that it could be repeated.	6
Study selection	9	State the process for selecting studies (i.e., screening, eligibility, included in systematic review, and, if applicable, included in the meta-analysis).	6–7
Data collection process	10	Describe method of data extraction from reports (e.g., piloted forms, independently, in duplicate) and any processes for obtaining and confirming data from investigators.	7
Data items	11	List and define all variables for which data were sought (e.g., PICOS, funding sources) and any assumptions and simplifications made.	4,7
Risk of bias in individual studies	12	Describe methods used for assessing risk of bias of individual studies (including specification of whether this was done at the study or outcome level), and how this information is to be used in any data synthesis.	7
Summary measures	13	State the principal summary measures (e.g., risk ratio, difference in means).	7
Synthesis of results	14	Describe the methods of handling data and combining results of studies, if done, including measures of consistency (e.g., I^2^) for each meta-analysis.	NA

From*:* Moher et al.^21^

### Appendix 2. Table of excluded studies.

**Table:**

**Citation**	**Reason for exclusion**
Ahmad, S., Hillyard, M., Bhatia, G., Rajenthran, S., & Davies, A. (2015). Five year progress and outcome for all patients assessed at the Charing Cross Gender Identity Clinic, London, UK, 2009. Transgender Health Care in Europe (EPATH 2015), Ghent, Belgium.	Existence or prevalence of (non-prescribed) hormone therapy use only
Bosom, M., & Medico, D. (2020). My first year on testosterone: Analyzing the trans experience through YouTube channels. Sexologies. doi: 10.1016/j.sexol.2020.10.001	No self-administration of hormone therapy
Calvar, C. E., Duran, Y., Cabrera, N. F., Dostal, D. J., Hirota, N. B., & Durante, M. I. (2018). Self-administration of cross-sex hormones in transsexual persons. High frequency of hyperprolactinemia. Endocrine Reviews, 39(2)	Existence or prevalence of (non-prescribed) hormone therapy use only
Coleman, E., Bockting, W., Botzer, M., Cohen-Kettenis, P., DeCuypere, G., Feldman, J., . . . Zucker, K. (2012). Standards of Care for the Health of Transsexual, Transgender, and Gender-Nonconforming People, Version 7. International Journal of Transgenderism, 13(4), 165–232. doi: 10.1080/15532739.2011.700873	Clinical standards of care
de Andrade, C. A. A., Loureiro, A. R., de Lima Neto, E. R., de Vasconcelos, E. M. R., & de Araújo, E. C. (2018). Self-care requirements of transsexual women using sex hormones, according to the Orem selfcare theory. Cogitare Enfermagem, 23(3), 85–94. doi: 10.5380/ce.v23i3.55748	Duplicate data (duplicate of de Andrade 2018, included in VP review)
Dendrinos, M. L., Budrys, N. M., & Sangha, R. (2019). Addressing the Needs of Transgender Patients: How Gynecologists Can Partner in Their Care. Obstet Gynecol Surv, 74(1), 33–39. doi: 10.1097/ogx.0000000000000633	Clinical standards of care
Feil, K., & Toth, B. (2020). The Transgender Clinic: What Needs to Be Considered?: Innsbruck Experiences Based on Case Studies. Journal fur Gynakologische Endokrinologie. doi: 10.1007/s41974-020-00146-8	No self-administration of hormone therapy
Fein, L. A., Salgado, C. J., & Estes, C. (2015). Transitioning transgender. Journal of Sexual Medicine, 12, 172	Duplicate data (duplicate of Fein 2017/earlier conference abstract)
Fein, L. A., Salgado, C. J., Alvarez, C. V., & Estes, C. M. (2017). Transitioning Transgender: Investigating the Important Aspects of the Transition: A Brief Report. International Journal of Sexual Health, 29(1), 80–88. doi: 10.1080/19317611.2016.1227013	No self-administration of hormone therapy
Feldman, J., & Safer, J. (2009). Hormone therapy in adults: suggested revisions to the sixth version of the Standards of Care. International Journal of Transgenderism, 11(3), 146–182. doi: 10.1080/15532730903383757	Clinical standards of care
Gooren, L., & Lips, P. (2014). Conjectures concerning cross-sex hormone treatment of aging transsexual persons. J Sex Med, 11(8), 2012-2019. doi: 10.1111/jsm.12563 10.1111/jsm.12563. Epub 2014 Apr 29.	Characteristics of hormone therapy users only
Gooren, L. J. (2014). Should cross-sex hormone treatment of transsexual subjects vary with ethnic group? Asian J Androl, 16(6), 809–810. doi: 10.4103/1008-682x.133972 10.4103/1008-682X.133972.	Characteristics of hormone therapy users only
Inman, E., Stelmak, D., Kobernik, E. K., Andino, J. J., Stroumsa, D., Moravek, M. B., & Randolph, J. F. (2020). Patient preferences for follow-up of gender affirming hormone therapy. Fertility and Sterility, 114(3), e69-e70. doi: 10.1016/j.fertnstert.2020.08.214	No self-administration of hormone therapy
Johnston, C. D., & Shearer, L. S. (2017). Internal Medicine Resident Attitudes, Prior Education, Comfort, and Knowledge Regarding Delivering Comprehensive Primary Care to Transgender Patients. Transgend Health, 2(1), 91–95. doi: 10.1089/trgh.2017.0007 10.1089/trgh.2017.0007. eCollection 2017.	No self-administration of hormone therapy
Khobzi Rotondi, N., Bauer, G. R., Scanlon, K., Kaay, M., Travers, R., & Travers, A. (2013). Nonprescribed Hormone Use and Self-Performed Surgeries: 'Do-It-Yourself' Transitions in Transgender Communities in Ontario, Canada. American Journal of Public Health, 103(10), 1830–1836. doi: 10.2105/AJPH.2013.301348	Existence or prevalence of (non-prescribed) hormone therapy use only
Liu, Y., Xin, Y., Qi, J., Wang, H., Hong, T., Yang, X., . . . Pan, B. (2020). The Desire and Status of Gender-Affirming Hormone Therapy and Surgery in Transgender Men and Women in China: A National Population Study. J Sex Med. doi: 10.1016/j.jsxm.2020.07.081	Existence or prevalence of (non-prescribed) hormone therapy use only
Majumder, A., & Sanyal, D. (2016). Outcome and preferences in female-to-male subjects with gender dysphoria: Experience from Eastern India. Indian J Endocrinol Metab, 20(3), 308–311. doi: 10.4103/2230-8210.179988	No self-administration of hormone therapy
Majumder, A., & Sanyal, D. (2017). Outcome and preferences in male-to-female subjects with gender dysphoria: Experience from Eastern India. Indian J Endocrinol Metab, 21(1), 21–25. doi: 10.4103/2230-8210.196000	No self-administration of hormone therapy (and duplicate of Majumder 2017)
Mepham, N., Bouman, W. P., Wylie, K., & Arcelus, J. (2015). People with gender dysphoria who self-prescribe cross sex hormones: prevalence, sources and side effect knowledge Transgender Health Care in Europe (EPATH 2015), Ghent, Belgium.	Existence or prevalence of (non-prescribed) hormone therapy use only
Riascos Sanchez, V., Pardo, P. J., Escobar, N., & Lamb Guerva, A. (2008). Peer-led assessment of transgender sex workers in Cali, Colombia. International AIDS Conference (AIDS 2008), Mexico City, Mexico.	Existence or prevalence of (non-prescribed) hormone therapy use only
Rotondi, N. K., Bauer, G. R., Scanlon, K., Kaay, M., Travers, R., & Travers, A. (2013). Nonprescribed Hormone Use and Self-Performed Surgeries: “Do-It-Yourself” Transitions in Transgender Communities in Ontario, Canada. American Journal of Public Health, 103(10), 1830–1836. doi: 10.2105/ajph.2013.301348	Existence or prevalence of (non-prescribed) hormone therapy use only (and duplicate of Khobzi Rotondi 2013)
Seal, L. (2015). Self-medication in trans people is not associated with deterioration in cardiovascular risk factors but is associated with reduced vitamin D levels and antidepressant use. Transgender Health Care in Europe (EPATH 2015), Ghent, Belgium.	Existence or prevalence of (non-prescribed) hormone therapy use only
Shires, D. A., Stroumsa, D., Jaffee, K. D., & Woodford, M. R. (2017). Primary care providers' willingness to continue gender-affirming hormone therapy for transgender patients. Fam Pract, 35(5), 576–581. doi: 10.1093/fampra/cmx119	No self-administration of hormone therapy
Onpanna, P., Daosodsai, P., Leelawat, K., & Porasuphatana, S. (2015). Cross-sex hormone use does not increase cardiovascular risk in young male-to-female transsexuals: A study of self-medication by healthy Thai cabaret dancers. Asian Biomedicine, 9(4), 511–518. doi: 10.5372/1905-7415.0904.421	Existence or prevalence of (non-prescribed) hormone therapy use only
Defreyne, J., Motmans, J., & T'Sjoen, G. (2017). Healthcare costs and quality of life outcomes following gender affirming surgery in trans men: a review. Expert Rev Pharmacoecon Outcomes Res, 17(6), 543–556. doi: 10.1080/14737167.2017.1388164	Existence or prevalence of (non-prescribed) hormone therapy use only
